# Hidden labour of caregiving for persons with epilepsy in Limpopo and Mpumalanga provinces, South Africa: a qualitative study

**DOI:** 10.3389/fpsyt.2026.1869112

**Published:** 2026-07-21

**Authors:** Happiness Ngobeni, Lufuno Makhado, Thendo Gertie Makhado

**Affiliations:** 1 Department of Public Health, Faculty of Health Sciences, University of Venda, Thohoyandou, South Africa; 2 Department of Advanced Nursing Sciences, Faculty of Health Sciences, University of Venda, Thohoyandou, South Africa

**Keywords:** caregiving labour, epilepsy caregiving, hidden labour, informal caregiving, rural health systems, unpaid care

## Abstract

Caregiving for persons with epilepsy is frequently conceptualised in terms of burden and stigma; however, such framings often obscure the labour-intensive nature of everyday caregiving practices. In rural South Africa, informal caregivers undertake extensive unpaid work that sustains the safety, well-being, and treatment continuity of persons with epilepsy. This study aimed to explore the hidden labour embedded in caregiving for persons with epilepsy in Limpopo and Mpumalanga Provinces. A qualitative exploratory-descriptive design was employed, involving in-depth semi-structured interviews with 60 caregivers of persons with epilepsy. Data were analysed using inductive thematic analysis, with interpretation guided by a labour-oriented analytical lens. The findings revealed three interconnected dimensions of hidden labour: (1) constant surveillance: continuous monitoring and caregiving readiness, characterised by continuous vigilance and caregiving demands; (2) protective labour: anticipating, preventing, and responding to seizure-related risk; and (3) sacrificial labour, involving restricted mobility, lost personal time, and constrained economic participation, where caregivers experienced reduced autonomy, limited social participation, and disruptions to employment and daily activities. These forms of labour were further intensified by socio-economic constraints, emotional strain, stigma, and limited access to formal health services. The study highlights caregiving as an invisible yet essential labour system that effectively substitutes formal healthcare within households. Recognising and supporting caregivers as key contributors to epilepsy care is critical for strengthening equitable and responsive health systems in resource-limited settings.

## 1 Introduction

In many rural settings, the day-to-day care of persons with epilepsy (PWE) is largely carried out within households rather than formal healthcare facilities (1). Family members often take primary responsibility for managing the condition, including supervising daily activities, responding to seizures, supporting treatment adherence, and assisting with healthcare visits (2, 3). These responsibilities extend beyond basic support and require continuous involvement in the health and well-being of the PWE. Existing studies on epilepsy caregiving have explored a range of experiences, including burden, stigma, emotional distress, supervision demands, household adjustments, and practical caregiving responsibilities (1, 2, 4). This literature has provided important insights into the everyday realities of supporting persons with epilepsy. However, caregiving is still rarely conceptualised explicitly as a form of labour embedded in everyday practices and in the functioning of the health system, particularly in rural, low-resource settings. As a result, the physical, supervisory, and organisational work undertaken by caregivers is often described but not examined as a form of hidden labour that sustains epilepsy care within households and communities. Although these perspectives highlight important challenges, they often present caregiving primarily in terms of negative experiences and may not fully capture the range of work that caregivers perform (2). In particular, they pay limited attention to the everyday physical, emotional, and organisational tasks necessary to sustain care within households.

The concept of hidden or invisible labour offers an alternative way to understand caregiving. Hidden labour refers to essential work that is often unpaid and not formally recognised within economic or health systems (5). In the study contexts, this includes the unpaid care work provided by family members that supports treatment and recovery outside formal institutions. Caregivers may take on roles that resemble those of healthcare providers, including monitoring symptoms, managing emergencies, and coordinating care, yet these contributions are not always explicitly acknowledged within health system structures (6).

In the context of epilepsy, caregiving demands are intensified by the unpredictable nature of seizures and the need for constant vigilance (3). Caregivers must be prepared to respond to sudden health events while also managing emotional strain and social challenges (7). These responsibilities are further shaped by broader structural factors such as poverty, limited access to healthcare services, and fragmented support systems in rural areas (8).

In South Africa, particularly in rural provinces such as Limpopo and Mpumalanga, caregivers often play a central role in supporting the ongoing care of persons with epilepsy within households and communities. Their responsibilities may include supervision, responding to seizures, promoting safety, and facilitating access to healthcare when required. However, the extent of these contributions is not always fully reflected in research and policy discussions on epilepsy care.

This study addresses this gap by examining caregiving through a labour-focused lens. Rather than viewing caregiving solely as a burden, the study explores it as a form of hidden labour embedded in everyday practices. Therefore, this study aims to explore the hidden labour involved in caregiving for persons with epilepsy in the Limpopo and Mpumalanga provinces of South Africa.

## 2 Methods

### 2.1 Study design

This study used a qualitative, exploratory-descriptive design to explore the hidden labour involved in caregiving among informal caregivers. A qualitative approach was suitable because it allowed for a deeper understanding of caregivers’ lived experiences, daily tasks, and the often unrecognised emotional, physical, and social demands of caregiving (9).

### 2.2 Study setting

This study was conducted in rural and peri-urban communities in Limpopo and Mpumalanga provinces of South Africa, specifically in Collins Chabane Local Municipality in the Vhembe District of Limpopo Province and Bushbuckridge Local Municipality in the Ehlanzeni District of Mpumalanga Province. These areas are predominantly rural and are characterised by high levels of poverty, unemployment, and socioeconomic vulnerability, with many households relying on social grants and informal sources of income (10). These communities are also characterised by limited formal employment opportunities and high dependence on informal, temporary, or grant-based income. This labour-market context is important for interpreting caregiving experiences because the requirement for caregivers to remain available at home may further constrain their ability to participate in already limited income-generating opportunities. Access to healthcare services is often constrained by shortages of healthcare personnel, limited specialist services, inadequate transport infrastructure, and long travel distances to health facilities.

The study sites were also particularly relevant because epilepsy is frequently understood and managed within broader cultural and traditional belief systems that may influence health-seeking behaviour, treatment adherence, family responses, and community perceptions of the condition. Previous research conducted in these provinces has shown that cultural, traditional, and faith-based understandings of epilepsy may shape how families interpret the condition, seek support, and combine different forms of care. These systems may provide social and spiritual support, although some beliefs may also contribute to stigma or delays in accessing biomedical services (11).

These contextual factors make Limpopo and Mpumalanga appropriate settings for exploring caregiving experiences because they shape the everyday responsibilities assumed by family caregivers, including supervision, seizure monitoring, risk prevention, treatment support, and navigating barriers to healthcare access. Examining caregiving within these settings, therefore, provides important insights into the often hidden and unpaid labour performed by families caring for relatives with epilepsy.

### 2.3 Population and sampling

The study population comprised informal caregivers of people with epilepsy (PWE). Informal caregivers were defined as adult family members or relatives who provided primary or substantial unpaid care to a person diagnosed with epilepsy and residing in the same household (12). Participants included parents, grandparents, spouses, siblings, and other family members actively involved in caregiving activities. Other relatives referred to extended family members, such as aunts, uncles, cousins, or in-laws, who lived with or near the person with epilepsy and were directly involved in daily caregiving. Daily caregiving included supervision, seizure monitoring, assistance during or after seizures, support with medication routines, accompanying the person to healthcare visits, and ensuring safety within the household. A combination of purposive and snowball sampling was used. Initial participants who met the inclusion criteria were purposively identified through community entry processes and local networks. These participants subsequently referred other eligible caregivers through snowball sampling.

To reduce the risk of homogeneity associated with snowball sampling, recruitment was monitored throughout data collection to ensure variation across key participant characteristics. These included province, gender, age group, caregiver relationship to the person with epilepsy, educational background, employment status, and duration of caregiving. Although referrals were used to identify additional eligible caregivers, the research team reviewed emerging sample characteristics during recruitment and prioritised participants who added variation to the sample. For example, recruitment sought to include caregivers from both Limpopo and Mpumalanga, male and female caregivers, and caregivers in different family caregiving roles, such as parents, siblings, spouses, and extended relatives, as well as caregivers with diverse employment and educational backgrounds. Recruitment also continued after early indications of saturation to confirm that the perspectives of caregivers from both provinces were adequately represented. This approach helped limit the tendency of snowball sampling to reproduce similar social networks and strengthened the sample’s diversity and transferability.

To be included in the study, participants had to be 18 years or older, live in the study area, and have provided ongoing care to a family member with epilepsy. Those who could not give written informed consent or who were not actively involved in caregiving were excluded. A total of 60 caregivers were interviewed, with 30 from Limpopo and 30 from Mpumalanga. The sociodemographic characteristics of the participants are presented in **Table 1**.

**Table 1:** Sociodemographic characteristics of participants by province.

Characteristic	Mpumalanga (n=30)	Limpopo (n=30)
Age 18–30 years	9	4
Age 31–50 years	15	18
Age 51 years and above	6	8
Male	10	4
Female	20	26
Single	22	17
Married	4	8
Divorced	0	2
Widowed	4	3
No formal education	4	1
Primary education	3	7
Secondary education	16	13
Tertiary education	7	9
Employed	5	7
Unemployed	22	15
Self-employed	1	5
Student	2	3
Sibling caregiver	11	13
Parent caregiver	8	8
Spouse caregiver	1	4
Other relatives	10	5
Caregiving duration: <1 year	1	1
Caregiving duration: 1–3 years	0	4
Caregiving duration: >3 years	29	25

Demographic information was collected during qualitative interviews to contextualise caregiving experiences.

### 2.4 Data collection procedure

Data were collected using in-depth semi-structured interviews conducted between December 2025 and February 2026. This method was selected because it allows participants to freely express their experiences while still ensuring that key topics are systematically explored (13). Semi-structured interviews are particularly valuable in qualitative research due to their flexibility, enabling researchers to probe responses and gain deeper insights while maintaining a consistent interview guide (14, 15). The interview guide included questions about caregiving duties, managing seizures, emotional and physical burden, community attitudes towards epilepsy, available support, coping strategies, and access to healthcare and services. See **Supplementary Material 2** for detailed questions on the interview guide. Interviews were conducted by the researcher (HN) in participants’ preferred languages, including Xitsonga, Tshivenda, Sepedi, and siSwati. Interviews took place in private, convenient locations, such as participants’ homes or other agreed-upon safe spaces. Each interview lasted between 40 and 65 minutes. With consent, interviews were audio-recorded and supported by field notes that captured non-verbal cues, context, and initial reflections. Recordings were transcribed word-for-word. Interviews conducted in local languages were translated into English by language experts at the University of Venda. To ensure accuracy and preserve the original meaning, the translated transcripts were cross-checked against the audio recordings. A total of 60 caregivers were interviewed, comprising 30 participants from Limpopo and 30 from Mpumalanga. Data saturation was assessed concurrently with data collection and analysis. Following each interview, transcripts were reviewed to identify emerging codes, patterns, and themes related to caregivers’ experiences. Saturation was considered reached when three consecutive interviews yielded no new themes or substantive insights relevant to the study objectives. Initial indications of saturation emerged before data collection was completed; however, recruitment continued to ensure adequate representation from both study provinces and to capture potential variations in caregiving experiences across the two settings. The final sample of 60 participants confirmed thematic saturation across both provinces and increased confidence that the range of caregivers’ experiences had been sufficiently captured. Thus, the final sample size was informed by both thematic saturation and the need to obtain perspectives from caregivers in each study site. Reflexivity was maintained throughout the research process. The researchers are public health and nursing researchers with experience working in rural South African communities and in epilepsy-related research. Although the researchers had prior experience conducting research in similar rural settings, they did not have pre-existing caregiving relationships with participants. To minimise potential bias during data collection and interpretation, reflexive note-taking, regular team discussions, and collaborative analysis were used to critically reflect on assumptions, participant interactions, and interpretation of findings. The final versions were subsequently reviewed by HN, LM, and TGM.

### 2.5 Data analysis

Data were analysed using inductive thematic analysis, a method that enables the identification and interpretation of patterns grounded in participants’ experiences (16, 17). This approach was selected for its flexibility and suitability for exploratory qualitative research (16). The analysis followed six phases as outlined by Braun and Clarke (16). First, the researchers familiarised themselves with the data by reading the transcripts multiple times. Second, initial codes were generated from meaningful segments of the data. Third, similar codes were organised into categories and potential themes. Fourth, these themes were reviewed and refined by comparing them against the dataset to ensure consistency and coherence (17). Fifth, themes were clearly defined and named. Finally, illustrative quotes were selected to support each theme.

Coding proceeded inductively and remained close to participants’ accounts in the initial stages. Examples of initial codes included “always watching,” “cannot leave the person alone,” “fear of sudden seizures,” “checking triggers,” “avoiding fire or cooking,” “rushing to hospital,” “unable to go to work,” “cannot run errands,” and “loss of personal time.” These initial codes were then compared across transcripts and grouped into broader categories. For example, codes relating to being physically present, watching throughout the day, and the inability to delegate care were grouped under the category “continuous supervision.” Codes relating to identifying triggers, preventing injury, restricting risky activities, and responding to emergencies were grouped under “risk anticipation and protection.” Codes relating to inability to work, restricted movement, loss of social participation, and time sacrificed for caregiving were grouped under “constraints on caregiver autonomy.” These categories were reviewed against the full dataset and refined into the final themes as shown in **Table 2**. The research team used ATLAS.ti Version 8.4 to organise codes and retrieve related data extracts. An independent co-coder reviewed selected transcripts and coding decisions, and differences were discussed until consensus was reached.

**Table 2:** Coding pathway from data extracts to final themes.

Data extract	Initial code	Category	Final theme
“You have to be present at all times.”	Always present; cannot leave	Continuous supervision	Constant surveillance: continuous monitoring and caregiving readiness
“The attacks don’t give any warning; they just happen suddenly.”	Sudden seizures; uncertainty	Continuous vigilance	Constant surveillance: continuous monitoring and caregiving readiness
*“Paying attention to triggers … people don’t notice.”*	Monitoring triggers	Risk anticipation	Protective labour: anticipating, preventing, and responding to seizure-related risk
*“We didn’t allow her to cook … she might fall into the fire.”*	Restricting risky activity; preventing injury	Environmental safety management	Protective labour: anticipating, preventing, and responding to seizure-related risk
*“I found him numb, foam coming out from his mouth … We rushed him to the hospital.”*	Emergency response	Crisis management	Protective labour: anticipating, preventing, and responding to seizure-related risk
*“I can’t even go to the store, I can’t even do anything.”*	Cannot leave home; loss of freedom	Restricted mobility	Sacrificial labour: restricted mobility, lost time, and constrained economic participation
*“I can’t even go to work … I have to come back on time.”*	Employment disruption; constant availability	Economic and time sacrifice	Sacrificial labour: restricted mobility, lost time, and constrained economic participation

To ensure consistency and enhance the trustworthiness of the analysis, the research team engaged an independent co-coder. Coding was conducted collaboratively, with discrepancies discussed and resolved through consensus until agreement was achieved, a process recommended to improve consistency and reliability in qualitative data analysis (18). Analysis included data from both provinces, with attention given to similarities and differences between Limpopo and Mpumalanga. Trustworthiness was ensured using the criteria of credibility, dependability, confirmability, and transferability (19). Credibility was strengthened through prolonged engagement with the data, the use of verbatim quotations, and regular discussions among the research team (17). Where applicable, member checking was conducted by clarifying issues during interviews or sharing summaries with selected participants (20). Dependability and confirmability were enhanced through maintaining an audit trail, reflexive note-taking, and collaborative coding discussions among the research team to ensure consistency and reduce researcher bias (17, 19). Transferability was addressed by providing detailed descriptions of the study context and participant characteristics to allow readers to assess the applicability of the findings to other settings (19).

### 2.6 Ethical considerations

Ethical approval was obtained from the University of Venda Human and Clinical Trials Research Ethics Committee (HCTREC) (Ethical Clearance No. FHS/25/PH/22/1811). Permission to conduct the study was also received from relevant traditional leaders and community authorities. Participation was voluntary, and written informed consent was obtained from all participants before data collection. Participants were informed about the purpose of the study, their right to refuse to answer questions, and their right to withdraw at any time without any consequences. Confidentiality and anonymity were ensured by removing identifying information from transcripts and using pseudonyms or codes. All data, including audio recordings, transcripts, and field notes, were stored securely and were only accessible to the research team.

## 3 Results

The final sample comprised 60 caregivers, with equal representation from Limpopo and Mpumalanga provinces (30 participants per province). The sample included variation in age, gender, marital status, educational attainment, employment status, caregiver relationship, and duration of caregiving, supporting diversity across key participant characteristics. Most participants were women; the largest age group was 31–50 years; and most had provided care for more than 3 years. Sibling caregivers formed the largest caregiving group, followed by parents, other relatives, and spouses. The full sociodemographic characteristics of participants are presented in **Table 1**. The predominance of women may reflect gendered caregiving expectations within the study communities; however, because snowball recruitment was partly referral-based, the gender composition may also have been influenced by participants’ referral networks.

The study analysis generated one overarching analytic pattern: caregiving as hyper-vigilant hidden labour. Hyper-vigilance permeated caregivers’ accounts because seizures were perceived as unpredictable and potentially harmful, even when they did not occur daily. Caregivers, therefore, described a need to remain alert, available, and prepared to intervene when necessary. However, this hyper-vigilance was expressed through three analytically distinct dimensions of labour. First, caregivers performed surveillance labour through continuous monitoring and 24-hour presence. Second, they performed preventive and protective labour by identifying triggers, modifying environments, restricting risky activities, and responding to emergencies. Third, they experienced mobility and time-sacrifice labour, as constant availability limited their ability to work, socialise, run errands, or attend to their own needs. These dimensions were therefore interconnected but not identical: the first concerned continuous monitoring, the second active risk prevention and emergency response, and the third the consequences of caregiving for caregivers’ autonomy, employment, and personal time.

### 3.1 Theme 1 – constant surveillance: continuous monitoring and caregiving readiness

Caregivers described caregiving as a continuous and demanding responsibility characterised by being consistently present, vigilant, and providing uninterrupted attention. This form of labour was experienced as ongoing and intensive, requiring caregivers to remain consistently alert due to the unpredictable nature of epilepsy. As a result, caregivers described caregiving as extending beyond routine support to involve a perceived need for continuous readiness and close supervision. Participants emphasised the necessity of always being physically present, highlighting the risks associated with even brief periods of absence. Furthermore, they also emphasised the need for sustained attention and proximity, with caregivers indicating that most of their time was devoted to monitoring and responding to potential seizures. The following quotations emerged:

“You have to be present at all times. You can’t delegate a child to look after them even for a few minutes because it is a very sensitive condition.” (Participant 2, Limpopo)

“It requires attention and time; you have to always be beside them 80% of the time, you have to devote it to her because she can have an episode anytime.” (Participant 14, Limpopo)

“It takes most of your time and attention. The way you should take care of them needs you to always monitor them. The attacks don’t give any warning; they just happen suddenly.” (Participant 10, Limpopo)

This constant state of alertness reflects the unpredictable onset of seizures, necessitating continuous observation and limiting opportunities for rest or disengagement. Similar experiences were reported by participants in Mpumalanga, who described caregiving as an all-encompassing responsibility that restricts personal autonomy and eliminates the possibility of leaving the person with epilepsy unattended. Within this context, some caregivers further highlighted how the demands of continuous supervision extended into broader areas of their lives, particularly affecting their ability to engage in employment and other income-generating activities. Collectively, these accounts illustrate caregiving as a form of constant surveillance labour embedded within the home, where caregivers assume uninterrupted responsibility for monitoring and responding to seizures. This underscores the hidden and intensive nature of caregiving, characterised by continuous vigilance, restricted autonomy, and the absence of clear boundaries between caregiving and personal time. The following narratives emerged:

“You always must observe them … the whole day…” (Participant 2, Mpumalanga)

“You can’t even take an hour without seeing them. You have to make sure you are always here with them.” (Participant 3, Mpumalanga)

“I can’t even go to work. Even the piece job that I get, I have to come back on time because I don’t know how they are when I’m not home.” (Participant 1, Mpumalanga)

### 3.2 Theme 2 – protective labour: anticipating, preventing, and responding to seizure-related risk

Caregiving also involved active preventive and protective labour, requiring caregivers to constantly anticipate, identify, and manage potential risks associated with epilepsy. This form of labour extended beyond supervision to include recognising seizure triggers, modifying the environment, and restricting certain daily activities to ensure safety. Participants highlighted the importance of being attentive to subtle triggers that could precipitate seizures, often requiring continuous monitoring of environmental conditions that may otherwise go unnoticed. The following quotation emerged:

“Paying attention to triggers. There are certain triggers that can cause the patient to have seizures, and people don’t notice.” (Participant 2, Limpopo)

“I’m now used to the fact that she is triggered by noise, so I ensure that they are always in a quiet environment.” (Participant 3, Limpopo)

“We didn’t allow her to cook. We were scared that if she cooks, she might fall into the fire.” (Participant 8, Limpopo)

“An epileptic person must never be alone … that’s very dangerous.” (Participant 6, Limpopo)

These accounts reflect how caregiving required continuous environmental control and precautionary decision-making to minimise harm, often limiting the independence of the person with epilepsy. In Mpumalanga, this protective labour extended further to include managing physical safety and behavioural instability during unpredictable seizure episodes, requiring caregivers to remain prepared to intervene at any moment. The following narratives emerged:

“He was falling … I got to see that the medical condition is severe.” (Participant 12, Mpumalanga)

“I rushed there, and I found him numb, foam coming out from his mouth, and he even peed on himself. We rushed him to the hospital…” (Participant 25, Mpumalanga)

These accounts demonstrate that preventive caregiving is not passive but rather an active and ongoing form of labour that involves constant risk assessment, environmental modification, and immediate responses to emergencies. This highlights the hidden complexity of caregiving, where caregivers must continuously anticipate danger and take proactive steps to protect the person with epilepsy.

### 3.3 Theme 3 – sacrificial labour: restricted mobility, lost time, and constrained economic participation

Caregivers described significant restrictions in their mobility and personal freedom, as caregiving responsibilities required constant proximity to the person with epilepsy. This resulted in a loss of independence, where everyday activities such as running errands, socialising, or attending to personal needs became difficult or impossible. Participants emphasised that caregiving limited their ability to move freely, as they were required to remain physically present and continuously available to respond to potential seizures. The following quotations emerged:

“The fact that I always have to be with the person with epilepsy 24/7. I can’t even go to the store, I can’t even do anything.” (Participant 15, Limpopo)

“It is very demanding because your movements are limited, you have to be careful always when you’re running errands or doing chores.” (Participant 3, Limpopo)

“It affects us because you always have to guard them.” (Participant 17, Limpopo)

These accounts illustrate how caregiving imposes constant spatial and temporal constraints, where caregivers’ movements are closely tied to the needs of the care recipient. As a result, caregivers experienced reduced autonomy and increased withdrawal from social and personal activities. Similar experiences were reported by participants in Mpumalanga, who described being largely confined to the home environment due to the unpredictability of seizures and the fear of leaving the person with epilepsy unattended. This restriction extended beyond the home into economic participation, where caregivers struggled to maintain employment because of the need for constant availability. The following narrative emerged:

“I can’t even go to work. Even the piece job that I get, I have to come back on time because I don’t know how they are when I’m not home.” (Participant 1, Mpumalanga)

Collectively, participants described caregiving as involving continuous supervision, proactive management of seizure-related risks, and ongoing availability to support persons with epilepsy. These responsibilities affected caregivers’ daily routines, mobility, employment, and participation in social activities.

## 4 Discussion

This study explored the hidden labour of caregiving among caregivers of people with epilepsy in Limpopo and Mpumalanga provinces, South Africa. The findings demonstrate that caregiving extends far beyond basic assistance, constituting a complex, continuous, and multidimensional form of labour embedded in daily practices of surveillance, risk management, and personal sacrifice. Importantly, this labour remains largely invisible because it is normalised within familial roles and situated within private household spaces, where it is neither formally recognised nor adequately supported. Although hyper-vigilance was the central mechanism linking the findings, the analysis shows that it operated through different forms of labour. Surveillance labour involved continuous observation; protective labour involved active risk prevention and emergency response; and sacrificial labour reflected the consequences of constant availability for caregivers’ mobility, employment, and personal time. Presenting the themes in this way clarifies that they are interconnected dimensions of hidden labour rather than three unrelated experiences.

A central finding of this study was the persistent surveillance and 24-hour responsibility required of caregivers. Participants described the need to remain available, alert, and ready because seizures could occur suddenly and without warning. This finding is consistent with epilepsy caregiving studies conducted in both high-income and low-resource settings. Smith et al. (21) identified “constant vigilance” as a key feature of caregiving in paediatric epilepsy, showing that caregivers often organise daily life around monitoring for possible seizures. Similarly, a qualitative study in the Netherlands found that parents caring for children with epilepsy valued seizure-detection devices because continuous supervision was emotionally demanding and linked to anxiety about seizure-related harm and sudden unexpected death in epilepsy (22). In Japan, caregivers of people with severe epilepsy syndromes also reported constant caregiving and lack of relief as major sources of burden (23). While previous epilepsy caregiving studies have documented constant vigilance and supervision, the present study extends this literature by conceptualising these activities as forms of hidden labour. Rather than viewing vigilance solely as a source of caregiver burden, the findings demonstrate how caregivers perform continuous monitoring work that is essential for preventing harm and maintaining the safety of persons with epilepsy. This perspective highlights the productive and health-sustaining functions of caregiving that are often overlooked in conventional burden-focused approaches.

The present findings also align strongly with evidence from low- and middle-income contexts. In Uganda, caregivers of persons with epilepsy reported that the condition required supervision, assistance, and continuous monitoring, particularly when seizures were unpredictable or associated with developmental difficulties (24). More recently, Nanziri et al. (25) reported that caregivers of children with epilepsy and/or cerebral palsy in southwestern Uganda experienced constant vigilance as necessary to prevent injury, reflecting similar concerns to those expressed by caregivers in Limpopo and Mpumalanga provinces, South Africa. A Malaysian qualitative study also found that caregiver burden among families of adults with epilepsy included ongoing monitoring responsibilities and restrictions on caregivers’ everyday lives (26). Taken together, these studies suggest that constant surveillance is a common feature of epilepsy caregiving across settings; however, its intensity may be heightened in under-resourced contexts where families have limited access to formal respite care, seizure-monitoring technologies, or community-based support. In Limpopo and Mpumalanga, responsibility for this intensive monitoring is largely absorbed within households, highlighting families’ central role in sustaining epilepsy care.

The finding of constant surveillance, however, should be interpreted carefully. The study does not suggest that all persons with epilepsy require continuous attendance or that seizures occur daily. Rather, caregivers described organising their lives around the perceived unpredictability of seizures and fear of seizure-related injury. In some cases, such vigilance may be protective, particularly where seizures are uncontrolled, severe, or occur in unsafe environments. However, constant supervision may also reflect caregiver anxiety, limited access to clinical guidance, and fear of social consequences. Excessive restriction may unintentionally reduce the autonomy of persons with epilepsy, reinforce dependency, and contribute to stigma. Therefore, caregiver support should not simply encourage constant monitoring, but should include education on seizure first aid, risk assessment, graded independence, and strategies that balance safety with the autonomy and dignity of persons with epilepsy.

The findings further highlight preventive and protective labour as a critical yet often overlooked dimension of caregiving. In this study, caregivers actively identified seizure triggers, modified home environments, and restricted potentially dangerous activities to minimise harm, demonstrating a continuous process of anticipation and risk management. This reflects embodied, experiential knowledge, in which caregivers develop practical expertise through lived experience and sustained interaction with the condition. These findings are consistent with qualitative research conducted across diverse settings. A meta-synthesis by Yu et al. (8) found that caregivers of children with epilepsy routinely engage in identifying seizure triggers, managing medication, and adapting the home environment to reduce risk. Similarly, Yang et al. (27) reported that caregivers play an active role in ongoing risk assessment and environmental control, often making daily decisions to prevent injury. Evidence from both high-income and low-resource contexts suggests that this form of labour is universal, although its intensity varies. For example, studies in high-income settings have shown that caregivers rely on structured self-management strategies and clinical guidance to manage triggers (28), whereas in low- and middle-income settings, caregivers rely more on informal knowledge and personal experience due to limited access to healthcare support (29). Although risk management has been reported in other studies of epilepsy caregiving, the findings from Limpopo and Mpumalanga illustrate how these responsibilities are shaped by rural resource constraints. Caregivers often relied on experiential knowledge and continuous personal supervision rather than external support services or assistive technologies. This suggests that preventive caregiving labour may become particularly intensive in settings where formal support systems are limited and responsibility for care is concentrated within households.

This type of labour extends beyond supervision to include anticipatory, interpretive, and decision-making processes, positioning caregivers as informal health practitioners within the home. Consistent with this, research has shown that caregivers of individuals with chronic conditions frequently assume roles comparable to “untrained health workers”, managing symptoms, administering care, and responding to emergencies without formal training (30). Despite the high level of skill and responsibility involved, such labour remains largely unrecognised within formal healthcare systems. This reflects broader structural inequalities between formal and informal care provision, where the burden of risk management is shifted to households without adequate support or compensation. In this context, preventive caregiving emerges as an active, skilled practice that involves ongoing risk assessment, decision-making, and emergency preparedness. A notable contribution of this study is the recognition that caregivers function as an extension of the epilepsy care system. Participants described activities that included continuous monitoring, assessing and managing seizure-related risks, responding to emergencies, and supporting ongoing care within the home. These responsibilities are largely performed outside formal healthcare settings and often without training, remuneration, or formal recognition. The findings, therefore, raise important questions about the extent to which health systems rely on informal caregiving labour while providing limited support to those who perform this work. In rural settings such as Limpopo and Mpumalanga, where access to healthcare services may be constrained, caregivers appear to play a critical role in sustaining epilepsy care and ensuring continuity of support between healthcare encounters.

This study also identifies restricted mobility and the loss of personal time as key dimensions of hidden caregiving labour. Caregivers were often unable to leave care recipients unattended, limiting their participation in employment, social activities, and community life. Similar patterns have been reported in epilepsy research, where caregiving is shaped by the unpredictability of seizures, requiring constant availability and disrupting daily routines (8, 26). This contributes to social isolation and reduced economic participation, outcomes also widely documented in broader caregiving literature (30). In low-resource settings, these constraints are further intensified by poverty and limited access to support services, reinforcing financial insecurity and dependence (25). The predominance of women in caregiving roles may also have important implications for social and economic inequality in rural communities. In contexts characterised by limited employment opportunities, the requirement for continuous caregiving may further constrain women’s participation in income-generating activities, potentially reinforcing cycles of economic dependence and vulnerability. The findings suggest that caregiving responsibilities are not experienced in isolation but are shaped by broader social and economic conditions that influence caregivers’ opportunities and wellbeing. Thus, caregiving labour intersects with broader gendered and structural inequalities in rural settings. The findings also highlight the gendered nature of caregiving labour. Most participants in this study were women, reflecting broader patterns in which unpaid caregiving responsibilities are disproportionately carried by women within households and communities. Similar studies have shown that caregiving is often socially constructed as a feminine responsibility, resulting in unequal emotional, economic, and social burdens for women (30, 31). From a labour perspective, these findings highlight the feminisation of unpaid caregiving work within rural households. The predominance of women in caregiving roles suggests that responsibility for epilepsy care is often shaped by gendered expectations regarding who should provide care within families (31). As a result, women may assume substantial caregiving responsibilities without financial compensation, formal recognition, or adequate support. This distribution of caregiving labour has important implications for gender equity, as time devoted to caregiving may reduce opportunities for education, employment, income generation, and participation in community life (31). In the present study, caregiving responsibilities frequently limited women’s mobility, employment opportunities, and personal autonomy, reinforcing existing gender inequalities and economic vulnerability in rural settings.

These findings suggest that caregiving responsibilities are frequently normalised as familial obligations despite requiring substantial knowledge, judgement, and effort (31). As a result, the complexity and intensity of caregiving remain largely unacknowledged, particularly in contexts where formal support systems are limited. The reliance on informal caregivers has important implications for health systems, as it shifts the burden of long-term care to households without adequate support. While caregivers play a critical role in managing chronic conditions, this model places them at risk of economic strain, psychological distress, and burnout (8).

This study underscores the need for greater recognition of caregivers’ contributions to epilepsy management and continuity of care. Caregivers in this study demonstrated high levels of responsibility, including continuous monitoring, risk assessment, decision-making, and emergency response. These findings align with the literature that positions caregivers as key actors in health systems, performing complex tasks traditionally associated with trained professionals (30). Recognising this complexity is critical for informing policy and practice. Interventions should include structured caregiver training, psychosocial support, financial assistance, and greater integration of caregivers into formal healthcare systems. Addressing the hidden labour of caregiving is essential not only for improving caregiver well-being but also for strengthening the overall quality and sustainability of epilepsy care.

## 5 Conclusions, limitations and recommendations

This study highlights that caregiving for people with epilepsy is not just support, but a form of continuous and demanding labour that remains largely hidden. Caregivers are required to constantly monitor, anticipate risk, and adjust their lives to ensure safety, often without rest or recognition. This everyday work, although essential, is normalised within families and therefore overlooked as “real” labour. The findings show that this hidden labour limits caregivers’ independence and shapes how they use their time, making it difficult for them to participate fully in social and economic life. In this way, caregiving is not only a personal responsibility but also a structural issue linked to how care is organised and valued in society. Recognising caregiving as labour is important. It shifts the focus from seeing caregivers as simply “helping” to understanding them as key contributors to health systems. Greater support, visibility, and inclusion of caregivers in care planning are needed to ensure that this work is not carried out alone or unnoticed. This study has several limitations that should be considered when interpreting the findings. First, the study was conducted in selected rural communities in Limpopo and Mpumalanga provinces, which may limit the transferability of the findings to other settings. Second, the caregiver sample was heterogeneous, including participants of different ages, family relationships, and caregiving roles, such as parents, siblings, spouses, and other relatives. These differences may have shaped caregiving experiences and perspectives in ways that were not specifically explored in the analysis. Although sociodemographic information, including employment status, education level, caregiving role, and age categories, was collected and presented in **Table 1**, more detailed socioeconomic indicators, such as household income, asset ownership, and formal socioeconomic classifications, were not collected. Consequently, the study was unable to examine how socioeconomic position may have influenced caregiving experiences.

In addition, the study focused primarily on caregivers’ experiences and therefore did not systematically collect detailed demographic and clinical information regarding the persons with epilepsy receiving care. Information such as age, seizure frequency and severity, level of functional dependence, and the presence of comorbid conditions was not available for analysis. These factors may substantially influence caregiving responsibilities and experiences and should be considered in future research. Finally, the use of snowball sampling may have introduced selection bias, as recruitment relied partly on participants’ social and community networks. Consequently, caregivers who were more socially isolated, less connected to community structures, or not linked to referral networks may have been underrepresented in the sample. These caregivers may experience different challenges, support needs, or caregiving responsibilities from those captured in this study. As a result, some perspectives and experiences may not have been fully represented in the findings.

## Data Availability

The raw data supporting the conclusions of this article will be made available by the authors, without undue reservation.
