# Supplementary material for: Hidden labour of caregiving for persons with epilepsy in Limpopo and Mpumalanga provinces, South Africa: a qualitative study
Source: Front Psychiatry. 2026 Jul 21;17:1869112. doi: 10.3389/fpsyt.2026.1869112 (PMC13434456; doi:10.3389/fpsyt.2026.1869112)
Supplement: Supplementary file 1 [file Supplementaryfile1.docx]

**Supplementary Material: Semi-Structured Interview Guide for Caregivers of People with Epilepsy**

**Opening Statement:**

"Thank you for taking the time to speak with me today. I’d like to talk with you about your experiences as a caregiver of someone with epilepsy. There are no right or wrong answers—what matters is your honest perspective. This conversation is confidential, and you may choose not to answer any question or stop the interview at any time. Do you have any questions before we begin?"

**Section A: Emotional and Physical Burden**

1. **Can you describe a typical day in your role as a caregiver for someone with epilepsy?**

*Probing questions:* What parts of caregiving do you find most demanding? How do you manage those responsibilities?

**Section B: Social and Cultural Perceptions**

2. **How is epilepsy generally understood or viewed in your community?**

*Probing question:* Are there common beliefs or misconceptions you’ve encountered?

**Section C: Challenges and Facilitators in Caregiving**

3. **What are some of the biggest challenges you face in caring for a person with epilepsy?**

*Probing question:* Are there any things that make caregiving easier for you?

**Section D: Stigma and Discrimination**

4. **Have you or the person you care for experienced any stigma or discrimination because of epilepsy?**

*Probing question:* How have these experiences affected you or your family?

**Section E: Coping Mechanisms and Support Systems**

5. **What kinds of support (emotional, social, or practical) do you have in your caregiving role?**

*Probing question:* What helps you cope with the stress or emotional toll of caregiving?

**Section F: Community Attitudes**

6. **How do you think your community treats people with epilepsy and their families?**

*Probing question:* Have you noticed any changes in attitudes over time?

**Closing Statement**

"Thank you very much for sharing your experiences. Is there anything else you’d like to add that we haven’t discussed? Your insights are very valuable for helping us understand the needs and challenges faced by caregivers like yourself."
